# Somatostatin Mitigates Gastric Mucosal Damage Induced by LPS in a Male Wistar Rat Model of Sepsis

**DOI:** 10.3390/biom15040508

**Published:** 2025-03-31

**Authors:** Sergio D. Paredes, Jorge Hernández-Cortés, Farzin Falahat, Lisa Rancan, Javier Arias-Díaz, Elena Vara

**Affiliations:** 1 Department of Physiology, School of Medicine, Complutense University of Madrid, Avda. Complutense, s/n, 28040 Madrid, Spain; 2 Department of Biochemistry and Molecular Biology, School of Medicine, Complutense University of Madrid, Avda. Complutense, s/n, 28040 Madrid, Spain; jorgehcortes@hotmail.com (J.H.-C.); lisaranc@ucm.es (L.R.); evaraami@ucm.es (E.V.); 3 Department of Surgery, School of Medicine, Complutense University of Madrid, Avda. Complutense, s/n, 28040 Madrid, Spain; ffalahat@ucm.es (F.F.); javierar@ucm.es (J.A.-D.); 4 Oral and Maxillofacial Surgery Service, Hospital Clínico San Carlos, 28040 Madrid, Spain

**Keywords:** somatostatin, gastric mucosa, LPS-induced sepsis, myeloperoxidase, phospholipase A2, malondialdehyde, thromboxane B2, leukotriene B4, prostaglandin I2, phosphatidylcholine

## Abstract

Upper gastrointestinal bleeding from erosive gastritis remains associated with high mortality in septic or postoperative patients. While stress ulcer bleeding has declined, it still occurs in septic patients and is considered a manifestation of intestinal failure within multi-organ failure syndrome. The integrity of the gastric mucosal barrier plays a crucial role in protection against this condition. Somatostatin (SS) appears as a biomolecule with cytoprotective properties. We aimed to investigate whether SS treatment protected the gastric mucosa in a rat model of lipopolysaccharide (LPS)-induced sepsis. Rats received LPS (10 mg/kg) intraperitoneally, followed by saline or SS (200 μg/kg; 5 mL/kg) treatment after 30 min (early treatment group) or 120 min (late treatment group). Control rats received only saline. Two hours after saline or SS administration (total procedure duration of 150 or 240 min), gastric lavage, gastric mucosa, and plasma samples were collected for analysis. SS treatment mitigated the LPS-induced gastric mucosal barrier disruption preserving phosphatidylcholine (PC) levels, as well as decreasing leukocyte infiltration marker myeloperoxidase (MPO), inflammation-related enzyme phospholipase A2 (PLA2), and lipid peroxidation indicator malondialdehyde (MDA). SS also reduced arachidonic acid-related metabolites thromboxane B2 (TXB2) and leukotriene B4 (LTB4) while increasing prostaglandin I2 (PGI2). SS treatment effectively maintained gastric mucosal integrity, reducing inflammation, and modulating arachidonic acid metabolites. These findings suggest that SS may serve as a therapeutic agent for preserving gastric mucosal integrity and reducing inflammation in LPS-induced gastric injury.

## 1. Introduction

Despite the advances made in antibiotic research and intensive care therapy, septic shock continues to be a primary cause of death in intensive care units [1]. The organ structures most frequently affected are the lungs, heart, kidneys, gastrointestinal tract, liver, central nervous system, and coagulation network. Death often occurs due to the complete failure of one or more of these systems.

The consequences of this multiple organ dysfunction include adult respiratory distress syndrome, myocardial disorders, acute kidney failure, upper gastrointestinal bleeding, liver failure, and disseminated intravascular coagulation [2]. Bleeding from acute gastric mucosal ulcerations or stress ulcers is a significant manifestation of intestinal failure within multiple organ failure syndrome and remains associated with high mortality in septic or postoperative patients [3]. In sepsis-related erosive gastritis, disruptions in the gastric mucosal barrier, particularly its lipid component phosphatidylcholine (PC), may play a crucial role [4].

Septic shock can result from infections caused by viruses, protozoa, fungi, and bacteria. Lipopolysaccharide (LPS), or endotoxin, is the primary component of the outer membrane of Gram-negative bacteria and plays a critical role in immune system activation as the most important surface antigen of these bacteria. In the context of septic shock, LPS is widely recognized as a key external factor in triggering this severe medical condition [5].

Some effects of endotoxins are mediated by lipid-derived compounds, such as arachidonic acid metabolites, a group of biologically active lipids known as eicosanoids. LPS stimulates macrophages to produce these metabolites through the cyclooxygenase pathway, generating prostaglandins and thromboxanes, or the lipoxygenase pathway, producing leukotrienes [6]. Increasingly, phospholipase A2 (PLA2), a key enzyme regulating arachidonic acid metabolites, is recognized for its critical role in the inflammatory response during sepsis [7]. This effect may be driven by the activation of PLA2, induced by cytokines or other mediators, highlighting its significant implications in septic states.

The activation of neutrophils via chemokine/cytokine receptors or toll-like receptor 9 triggers the release of myeloperoxidase (MPO) from primary granules. MPO then facilitates the localized production of highly toxic oxidants, which play a role in causing tissue damage [8]. LPS in circulation triggers the secretion of various mediators that ultimately activate neutrophils, leading to the release of reactive oxygen species (ROS). Normally, the harmful effects of ROS are controlled by an antioxidant defense system; however, tissue damage occurs when ROS production becomes excessive and/or antioxidant defenses are diminished. This imbalance is common in conditions like severe sepsis [9]. In such cases, tissue destruction can be assessed by measuring the byproducts of lipid peroxidation in ischemic tissues, such as malondialdehyde (MDA).

On the other hand, in conditions such as septic shock, a massive release of nitric oxide (NO) occurs in the vascular wall, contributing to hypotension and reduced responsiveness to vasoconstrictors [10]. NO appears to mediate its effects, in part, through the activation of guanylate cyclase, leading to increased levels of cyclic guanosine monophosphate (cGMP). Similarly, carbon monoxide (CO) can function as an intracellular messenger, activating the soluble form of guanylate cyclase and exhibiting properties akin to those of NO.

Given that, during the systemic inflammatory response to infection, LPS interacts with immune cells to trigger a cascade of reactions that can ultimately result in multiple organ failure and death, efforts to interrupt this process using cytoprotective biomolecules are well-justified. Somatostatin (SS), a peptide hormone, has been extensively studied for its cytoprotective properties across various organ systems, including its protective effects observed in experimental models of liver and lung lesions, and pancreatitis, suggesting mechanisms that extend beyond its hormonal activity [11,12,13]. In addition, gastric ulceration has been reported to be alleviated by administration of the synthetic somatostatin analogue octreotide [14].

Based on the aforementioned points, the potential protective effect of SS on gastric mucosal alterations induced by LPS was investigated in the present study, with the aim to assess whether SS could reduce the damage caused by LPS in an experimental model of sepsis and explore its role in modulating LPS-triggered mediators that may contribute to its possible protective action.

## 2. Materials and Methods

### 2.1. Animals

Male Wistar rats from Janvier Labs (Le Genest-Saint-Isle, France), aged 2 months, and weighing 250–300 g, were housed in a controlled environment with automated light-dark cycles (12 h of light from 8:00 to 20:00, and 12 h of darkness) and maintained at a temperature of 22 ± 2 °C. The animals were provided with a standard diet from Pan-lab (Barcelona, Spain) and had access to water ad libitum. Humane care was ensured for all animals, and the study strictly adhered to the Ethical Norms for Animal Research as outlined by the European Union directive (2010/63/EU).

### 2.2. Experimental Design and Sample Collection

Experiments were conducted after the acclimation period, which allowed the animals time to stabilize in their new environment. A sample size calculator was used to compute the necessary samples to meet the desired statistical constraints. Random assignment was used to minimize potential confounders. No rats showed signs of exclusion criteria (signs of pre-existing illness, injury, or abnormal physiological conditions prior to the induction of sepsis using LPS).

We used a previously established sepsis model, chosen for its technical simplicity and reproducibility [15]. After the rats were fasted overnight, they were administered *Escherichia coli* 055:BS LPS (Sigma Chemical Co., St Louis, MO, USA) at a dose of 10 mg/kg b.w. intraperitoneally.

Thirty min (10 rats/group; early treatment group) or 120 min (10 rats/group; late treatment group) after LPS administration, the rats were randomly assigned to two sub-groups. Each sub-group received an intraperitoneal injection of tritiated choline ([methyl-^3^H] choline, 20 μCi; Sigma Chemical Co., St Louis, MO, USA) as a marker of PC synthesis, along with one of the following treatments: (1) saline at a dose of 5 mL/kg (LPS + Saline groups) or (2) SS (Sigma Chemical Co., St Louis, MO, USA) at a dose of 200 μg/kg; 5 mL/kg (LPS + SS groups). Two hours after the administration of tritiated choline with saline or SS treatments (i.e., 150 or 240 min post-LPS administration), the rats were anesthetized with ketamine (10 mg/kg; 2 mL/kg) injected intraperitoneally. Blood, gastric lavage, and gastric mucosa samples were then collected.

The animals in the control groups were treated identically to those in the experimental groups, receiving saline instead of LPS or SS.

To collect the gastric lavage samples, a laparotomy was performed, and the stomach was carefully dissected. The pylorus was ligated, and after cutting the esophago-gastric junction, a 14-gauge Teflon catheter connected to a syringe was inserted through it. The gastric cavity was then rinsed with 2 mL of 0.9% NaCl solution (at 4 °C). The recovered liquid was collected using the same syringe and subsequently frozen at −80 °C until the analysis of its NO_2_^−^ + NO_3_^−^, CO, and protein content.

For the gastric mucosa analysis, the stomach was fully removed and opened longitudinally along its lesser curvature. The gastric mucosa was then scraped using a curette, separated into several tubes, and stored at −80 °C until the measurement of the various parameters under investigation. Specifically, samples were collected from the corpus region of the glandular stomach. This region was chosen because it is the main site of gastric acid secretion and contains a high density of mucosal phospholipids, particularly PC, which is critical for maintaining barrier integrity. Additionally, the corpus is rich in D cells, which produce endogenous SS, making it a relevant site for investigating the protective effects of exogenous SS in the context of LPS-induced gastric injury. Unlike the pyloric and cardiac regions, which have distinct cellular compositions and functions, the corpus is more susceptible to sepsis-induced damage, allowing us to assess the potential of SS in preserving mucosal integrity under these conditions.

No adverse effects were observed during the entire experimental procedure.

### 2.3. Somatostatin (SS)

SS was measured using a radioimmunoassay (RIA). The buffer used for the SS RIA had the following composition: Na_2_HPO_4_ (0.04 mol/L), NaCl (0.14 mol/L), EDTA (0.025 mol/L), bovine albumin (0.25%), and a pH of 7.4. In each 4-mL tube, 100 µL of diluted SS-specific antibody, 500 µL of Na^125^I-labeled SS, and 100 µL of either the sample or SS standard solutions were added and incubated at 4 °C for 48 h. To separate the free SS from that bound to the antibody, 1 mL of a carbon-dextran suspension (0.25% carbon and 0.025% dextran) in phosphate buffer containing 0.9% NaCl was added to all tubes, except those containing only total activity. The radioactivity of the precipitate was then measured using a gamma particle counter. The sensitivity of the RIA was 0.03202 pg/tube, with the minimal error zone corresponding to a concentration range between 10 and 80 pg/tube. A specific in-house antibody [16], diluted to a final concentration of 1/56,000, was used for all determinations.

### 2.4. Phosphatidylcholine (PC) and Dipalmitoylphosphatidylcholine (DPPC)

To investigate the impact of bacterial LPS on PC synthesis in gastric mucosal cells, the incorporation of labeled choline ([methyl-^3^H] choline) into PC extracted from gastric tissue was measured. For PC extraction, a mucosal sample was homogenized in chloroform/methanol (2:1) at a final dilution of 1/17. The mixture was equilibrated at room temperature for 1 h, then filtered into a ground-glass tube with a stopper. The crude extract was combined with 0.2 times its volume of Folch’s solution, and the mixture was separated into two phases by centrifugation. After aspirating the upper phase, three washes were performed with UPS (50% of the original volume), centrifuging after each wash and discarding the upper phase. Finally, the lower phase and any remaining upper phase were combined into one phase by adding methanol, and the solution was evaporated under vacuum. The dried extract was re-dissolved in chloroform/methanol, transferred to a scintillation vial, and the radioactivity was measured using a liquid scintillation counter. Results were expressed as counts per minute (cpm)/mg protein.

Given that the PC fraction of the gastric mucosal barrier is predominantly composed of the disaturated form, DPPC, we next aimed to determine whether changes in overall PC labeling corresponded to alterations in the labeling of the saturated form. To isolate DPPC, the entire PC fraction was reacted with osmium tetroxide in carbon tetrachloride. The disaturated PC species were then separated from the unsaturated ones using thin-layer chromatography on boric acid-impregnated silica gel plates, employing a mixture of chloroform/methanol/ammonium chloride/H_2_O (75:25:1:2 *v*/*v*). For comparison, varying amounts of a standard DPPC solution were applied directly to the plate. This procedure was repeated to determine the recovery of disaturated species from the sample containing radioactive saturated PC. The recovery rate was 79.5 ± 6.2% (n = 6).

### 2.5. Myeloperoxidase (MPO), Phospholipase A2 (PLA2), and Malondialdehyde (MDA)

MPO content in gastric tissue was measured to assess the degree of leukocyte infiltration, using the modified method of Bradley [17]. Briefly, a sample of gastric mucosa was homogenized in phosphate buffer (pH 6.0) and centrifuged at 20,000× *g* for 15 min. The supernatant was discarded, and the pellet was resuspended in phosphate buffer containing hexadecyltrimethylammonium bromide to eliminate potential pseudoperoxidase activity and solubilize membrane-bound MPO. The suspension was sonicated for 20 s and subjected to three freeze-thaw cycles. An aliquot of the final supernatant was mixed with phosphate buffer containing O-dianisidine dihydrochloride and hydrogen peroxide, and its absorbance was measured at 460 nm.

PLA2 plays a key role in mediating inflammatory responses during sepsis, as bacterial LPS activates PLA2 in cell membranes, leading to phospholipid hydrolysis, a critical step in arachidonic acid release; thus, we assessed its activity in the gastric mucosa. PLA2 activity was measured by assessing ^3^H-arachidonate release using a suspension of *Escherichia coli* labeled with ^3^H-arachidonate as the substrate [18]. After homogenizing the tissue in phosphate-buffered saline (PBS) with 0.1 mmol/L phenylmethylsulfonylfluoride, 200 µL aliquots of the samples were incubated at 37 °C for 1 h in a buffer containing 0.1% Triton X-100, 40 mmol Trizma base, 0.65 mmol/L deoxycholic acid, 2 mmol/L CaCl_2_, and 0.25 µCi of the labeled *E. coli* suspension at pH 7.5. The reaction was terminated by adding 2 mL of 2-propanol/n-heptane/1 mol/L H_2_SO_4_ (40:10:1). Next, 2 mL of n-heptane and 3 mL of distilled water were added to form two phases, and the radioactivity of the upper phase was measured using a liquid scintillation counter. PLA2 activity was expressed as IU/mg protein, where one unit is equivalent to 1% of the total counts.

The production of MDA in the gastric mucosa was quantified as an indicator of lipid peroxidation, a process attributed to ROS generated by polymorphonuclear leukocytes, by means of the thiobarbituric acid (TBA) reaction. The sample was homogenized in PBS, and the resulting suspension was centrifuged at 3000× *g* for 10 min. An aliquot of the supernatant was collected, deproteinized, and allowed to react for 12 h with a solution containing hydrochloric acid (0.25 mol/L), trichloroacetic acid (15%), thiobarbituric acid (3 mmol/L), and 2-tetrabutyryl-4-methylphenol (0.1%). After the 12-h reaction, its absorbance was measured at 533 nm, using 1-1-3-3-tetraethoxypropane as the standard.

### 2.6. Arachidonic Acid Metabolites: Prostaglandin E2 (PGE2), Prostaglandin I2 (PGI2), Thromboxane B2 (TXB2), and Leukotriene B4 (LTB4)

The gastric mucosa releases a variety of mediators that can either protect or worsen tissue damage, including arachidonic acid metabolites, which are thought to play a role in the development of stress ulcers. PGE2 is recognized for its cytoprotective effect in the gastric mucosa, while PGI2 and TXB2, both derived from the cyclooxygenase pathway, have opposing effects. TXB2, the stable metabolite of thromboxane A2 (TXA2), acts as a vasoconstrictor and promotes platelet aggregation, whereas PGI2 has a vasodilatory effect and inhibits platelet aggregation. Arachidonic acid can also be metabolized through alternative pathways to cyclooxygenase, including the lipoxygenase pathway. Considering the recognized role of leukotrienes in the development of stress ulcers, which may contribute to gastric mucosa damage, potentially by promoting ROS production and enhancing neutrophil chemotactic and adhesion activity, it was furthermore examined whether LPS affected LTB4 levels in the gastric mucosa. Measurements were performed using specific RIA kits (Amersham, UK). Following the specific extraction, all samples were processed immediately after collection. PGE2 was converted into its methyl oxymate derivative for analysis. Due to its instability, PGI2 was quantified by measuring the concentration of its stable metabolite 6-keto-PGF1α.

### 2.7. Nitric Oxide (NO), Nitrate Synthase (NOS) Activity, and Nitrosothiols

NO was quantified as nitrite (NO_2_^−^) content. To do this, the samples were deproteinized by adding sulfosalicylic acid and incubating for 30 min at 40 °C. After centrifugation for 20 min (12,000× g), the supernatant was separated and incubated with nitrate reductase to reduce NO_3_^−^ to NO_2_^−^. Griess reagent was then added to all samples, and their absorbance was measured at 546 nm, using a NaNO_2_ solution as the standard.

NOS activity was assessed by measuring the conversion of ^14^C-arginine to citrulline. Tissue samples were homogenized in a buffer containing 10^−2^ mol/L Hepes, 0.32 mol/L sucrose, 10^−4^ mol/L EDTA, 10^−3^ mol/L dithiothreitol, 10 µg/mL leupeptin, 2 µg/mL aprotinin, and 1 mg/mL phenylmethanesulfonylfluoride. The homogenates were then centrifuged at 100,000× *g* at 40 °C for 20 min, and an aliquot of the supernatant was incubated with the radioactive precursor for 30 min at 37 °C. After purification by ion exchange on a Dowex AG 8 resin column, radioactivity was measured using a liquid scintillation counter.

Since some of the biological effects of NO may be mediated indirectly through carrier molecules such as nitrosothiols (NO + NOSH), their concentration was measured with the Saville method [19] to rule out the possibility of increased NO production that may have gone undetected due to its presence in this biological form. The Saville method is based on the binding of the Hg^2+^ cation to S, forming a complex susceptible to nucleophilic attack by H_2_O molecules.

### 2.8. Carbon Monoxide (CO)

To measure the amount of CO produced, hemoglobin (Hb), which binds to CO, was added to the samples, and the carboxyhemoglobin (COHb) level was determined. After adding Hb, the samples were allowed to incubate for 1 min to ensure complete CO binding. They were then diluted with a phosphate buffer containing sodium dithionite and incubated for 10 min at room temperature. Absorbance was measured at 421 and 432 nm, with a buffer-only sample used as the blank.

### 2.9. Cyclic Guanosine Monophosphate (cGMP)

cGMP was measured using specific RIA kits (NEN, Boston, MA, USA). Briefly, isomethyl butyl xanthine (a phosphodiesterase inhibitor) was added, and the tissue was homogenized manually in a glass homogenizer before being sonicated with an ultrasound disruptor. Proteins were then precipitated, and the cyclic nucleotide was extracted using 80% ethanol (*v*/*v*). After centrifugation, the supernatant was divided into two aliquots, transferred to RIA tubes, and evaporated at 37 °C. The residues were reconstituted with RIA buffer and quantified using RIA according to the specific instructions for each kit. The recovery of [^3^H] cGMP was 97.6 ± 2.1% (n = 6).

### 2.10. Proteins

Protein concentration was determined using the colorimetric method described by Bradford [20]. This method is based on the binding of Coomassie Brilliant Blue dye to proteins, which causes a shift in the absorption maximum of the dye from 465 nm to 595 nm. The absorbance of the samples at 595 nm was measured and compared to a standard curve. The protein-dye complex has a high extinction coefficient, ensuring high sensitivity in protein measurement.

### 2.11. Statistical Analysis

Nonparametric statistical tests were applied. The data are presented as mean ± SEM. To compare groups, the Kruskal-Wallis test for analysis of variance (ANOVA) by ranks was used, followed by the Mann-Whitney test for independent samples to determine the source of the differences if the results were significant. Confidence levels of 95% (*p* < 0.05) and 99% (*p* < 0.01) were considered statistically significant and highly significant, respectively. Statistics were calculated using SigmaPlot v12.3 (Systat Software, Inc., San Jose, CA, USA) and Prism v9 (GraphPad Software, Inc., Boston, MA, USA).

## 3. Results

As all experiments were conducted simultaneously and synchronously under identical environmental conditions, including housing, feeding, light cycles, and handling, as in [15], single untreated control or saline/LPS-challenged groups were used for both. Since they were not subjected to any experimental treatment, they represent, respectively, a true physiological or pathophysiological baseline for experimental groups. This approach aligns with the 3Rs principle (Replacement, Reduction, Refinement) by minimizing unnecessary and redundant animal use while maintaining scientific rigor. Additionally, treatments were administered to separate groups, ensuring no pharmacological interaction between them. Statistical analysis was conducted appropriately to account for multiple comparisons and validate the use of shared controls. Given these considerations, using common control groups provided a reliable reference for both treatments while ensuring methodological consistency and ethical responsibility.

### 3.1. Effect of Early and Late SS Treatment on SS, PC, and DPPC Content in Gastric Mucosa After LPS Administration

**Figure 1:**
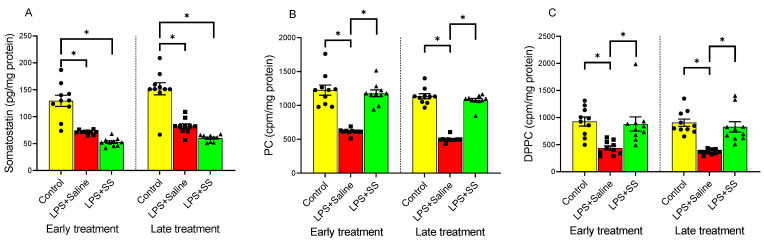
The figure shows the content of SS (pg/mg protein) (**A**), PC (cpm/mg protein) (**B**) and DPPC (cpm/mg protein) (**C**) in gastric mucosa of male Wistar rats after 30 min of LPS administration + 120 min of SS treatment (early treatment; total procedure duration of 150 min) or 120 min of LPS administration + 120 min of SS treatment (late treatment; total procedure duration of 240 min). Treated animals received LPS + Saline (red columns) or LPS + SS (green columns). Control animals received only saline (yellow columns). (*) *p* < 0.01 ((**A**): Control vs. the rest of the groups; (**B**,**C**): LPS + Saline vs. the rest of the groups).

SS plays a crucial role in regulating gastric acid secretion and maintaining mucosal integrity, protecting the gastric mucosa by reducing inflammation and promoting tissue repair, which is essential for healing ulcers and preventing further damage. PC helps preserve the protective mucosal barrier and modulates inflammatory responses, safeguarding the stomach lining. DPPC, like other forms of PC, contributes to this function. Figure 1 shows the effect of LPS on SS (Figure 1A), PC (Figure 1B), and DPPC (Figure 1C) content in the gastric mucosa. Compared to the control group, intraperitoneal injection of LPS significantly decreased SS levels in the gastric mucosa (control group 129.4 ± 10.40 vs. LPS + Saline early treatment group 71.23 ± 1.195 pg/mg protein, and control group 151.8 ± 11.26 vs. LPS + Saline late treatment group 81.98 ± 4.45 pg/mg protein, *p* < 0.01; Figure 1A), as well as induced a significant decrease in the incorporation of labeled choline into PC (control group 1226 ± 74.84 vs. LPS + Saline early treatment group 613.1 ± 13.63 cpm/mg protein, and control group 1135 ± 37.73 vs. LPS + Saline late treatment group 499.7 ± 13.46 cpm/mg protein, *p* < 0.01; Figure 1B), in addition to reducing DPPC synthesis by mucosal cells (control group 925.4 ± 83.58 vs. LPS + Saline early treatment group 437.3 ± 39.29 cpm/mg protein, and control group 905.1 ± 67.41 vs. LPS + Saline late treatment group 366.4 ± 14.44 cpm/mg protein, *p* < 0.01; Figure 1C).

The introduction of SS, both 30 and 120 min after LPS administration, did not reverse the effect produced by LPS in gastric mucosa SS content, whose levels remained significantly reduced compared to the controls (LPS + SS early treatment 52.62 ± 2.37 vs. control group 129.4 ± 10.40 pg/mg protein, *p* < 0.01; LPS + SS late treatment 60.07 ± 1.93 vs. control group 151.8 ± 11.26 pg/mg protein, *p* < 0.01; Figure 1A).

Treatment with SS significantly increased the incorporation of labeled choline into gastric mucosal PC, both at 150 min (LPS + SS 1179 ± 49.52 vs. LPS + Saline 613.1 ± 13.63 cpm/mg protein, *p* < 0.01, early treatment) and at 240 min (LPS + SS 1075 ± 27.58 vs. LPS + Saline 499.7 ± 13.46 cpm/mg protein, *p* < 0.01, late treatment), thus counteracting the effect produced by LPS, returning PC levels to those observed in the control group (Figure 1B).

Treatment with SS had a similar effect on DPPC as seen with PC, both at 30 min (early treatment group) and 120 min (late treatment group) after LPS administration (LPS + SS 881.1 ± 130.6 vs. LPS + Saline 437.3 ± 39.29 cpm/mg protein, *p* < 0.01, and LPS + SS 827.4 ± 95.54 vs. LPS + Saline 366.4 ± 14.44 cpm/mg protein, *p* < 0.01, at 150 min and 240 min, respectively), effectively blocking the LPS-induced effect, with no significant differences compared to the control group (Figure 1C).

In summary, LPS administration significantly reduced SS levels, PC incorporation, and DPPC synthesis in the gastric mucosa compared to the control groups. SS treatment, administered both early and late after LPS injection, did not reverse the LPS-induced decrease in SS levels but did significantly increase the incorporation of labeled choline into PC and restore DPPC synthesis, counteracting the effects of LPS and returning these levels to those observed in the control groups.

### 3.2. Effect of Early and Late SS Treatment on MPO Activity, PLA2 Activity, and MDA Content in Gastric Mucosa After LPS Administration

**Figure 2:**
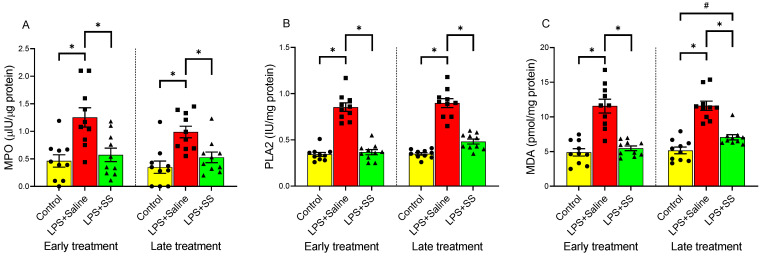
The figure shows the content of MPO activity (µIU/µg protein) (**A**), PLA2 activity (IU/mg protein) (**B**), and MDA content (pmol/mg protein) (**C**) in the gastric mucosa of male Wistar rats after 30 min of LPS administration + 120 min of SS treatment (early treatment; total procedure duration of 150 min) or 120 min of LPS administration + 120 min of SS treatment (late treatment; total procedure duration of 240 min). Treated animals received LPS + Saline (red columns) or LPS + SS (green columns). Control animals received only saline (yellow columns). (*) *p* < 0.01 LPS + Saline vs. the rest of the groups; (#) *p* < 0.05 LPS + SS vs. the control group.

MPO is an enzyme found in neutrophils that plays a key role in the immune response by producing ROS to kill pathogens. Quantification of MPO is commonly used as a marker of neutrophil infiltration to assess the extent of mucosal inflammation and injury. LPS administration caused a significant increase in MPO (1.25 ± 0.17 vs. 0.46 ± 0.11 µUI/µg protein, *p* < 0.01; Figure 2A), PLA2 (0.85 ± 0.05 vs. 0.34 ± 0.02 IU/mg protein, *p* < 0.01; Figure 2B), and MDA (11.57 ± 0.997 vs. 4.88 ± 0.53 pmol/mg protein, *p* < 0.01; Figure 2C) at 150 min (early treatment) compared to the control group. Similarly, at 240 min (late treatment), a significant increase was also observed in MPO activity in the gastric mucosa (0.99 ± 0.11 vs. 0.35 ± 0.11 µUI/µg protein, *p* < 0.01; Figure 2A), PLA2 activity (0.9 ± 0.05 vs. 0.35 ± 0.014 IU/mg protein, *p* < 0.01; Figure 2B), and MDA production (11.61 ± 0.67 vs. 5.16 ± 0.48 pmol/mg protein, *p* < 0.01; Figure 2C).

Administration of SS, both 30 min (early treatment) and 120 min (late treatment) after the intraperitoneal injection of LPS, caused a significant decrease in MPO activity in the gastric tissue compared to the LPS group (LPS + SS 0.57 ± 0.12 vs. LPS + Saline 1.25 ± 0.17 µUI/µg protein, *p* < 0.01; and LPS + SS 0.53 ± 0.096 vs. LPS + Saline 0.99 ± 0.11 µUI/µg protein, *p* < 0.01; Figure 2A), with values obtained after SS treatment similar to those observed in the control group.

PLA2 is an enzyme that releases free fatty acids, such as arachidonic acid, and lysophospholipids, playing a key role in membrane remodeling, inflammation, and the production of bioactive lipid mediators like prostaglandins and leukotrienes, influencing gastric mucosal defense, ulcer formation, and overall gastric pathology. Treatment with SS, both 30 min (early treatment) and 120 min (late treatment) after LPS administration (total procedure duration of 120 min and 240 min, respectively), also fully reversed the effect of LPS (LPS + SS 0.37 ± 0.028 vs. LPS + Saline 0.85 ± 0.05 IU/mg protein, *p* < 0.01; and LPS + SS 0.482 ± 0.02 vs. LPS + Saline 0.9 ± 0.05 IU/mg protein, *p* < 0.01; Figure 2B), with no significant differences compared to the control groups.

Finally, MDA is primarily generated as a byproduct of lipid peroxidation, where ROS degrade polyunsaturated fatty acids. It promotes oxidative stress and inflammation. In the context of the gastric mucosa, it seems to be linked to mucosal damage in the gastric lining, weakened defenses, increased ulcer risk, and disease severity. As observed for MPO and PLA2, treatment with SS 30 min after LPS administration (early treatment) completely blocked the effect of LPS on MDA production (LPS + SS 5.47 ± 0.35 vs. LPS + Saline 11.57 ± 0.997 pmol/mg protein, *p* < 0.01; Figure 2C). However, the late treatment (120 min after LPS administration; total procedure duration of 240 min) only partially reversed the effect of LPS (LPS + SS 7.06 ± 0.39 vs. LPS + Saline 11.61 ± 0.67 pmol/mg protein, *p* < 0.01), with values significantly higher than the control (LPS + SS 7.06 ± 0.39 vs. control group 5.16 ± 0.48 pmol/mg protein, *p* < 0.05; Figure 2C).

Taken together, the results showed that LPS administration significantly increased MPO, PLA2, and MDA levels, indicating heightened inflammation and oxidative stress in the gastric mucosa. In general, both early (30 min after LPS; total procedure duration of 120 min) and late (120 min after LPS; total procedure duration of 240 min) treatments with SS effectively reduced these markers to near control levels.

### 3.3. Effect of Early and Late SS Treatment on Arachidonic Acid Metabolites PGE2, PGI2, TXB2, and LTB4 in Gastric Mucosa After LPS Administration

**Figure 3:**
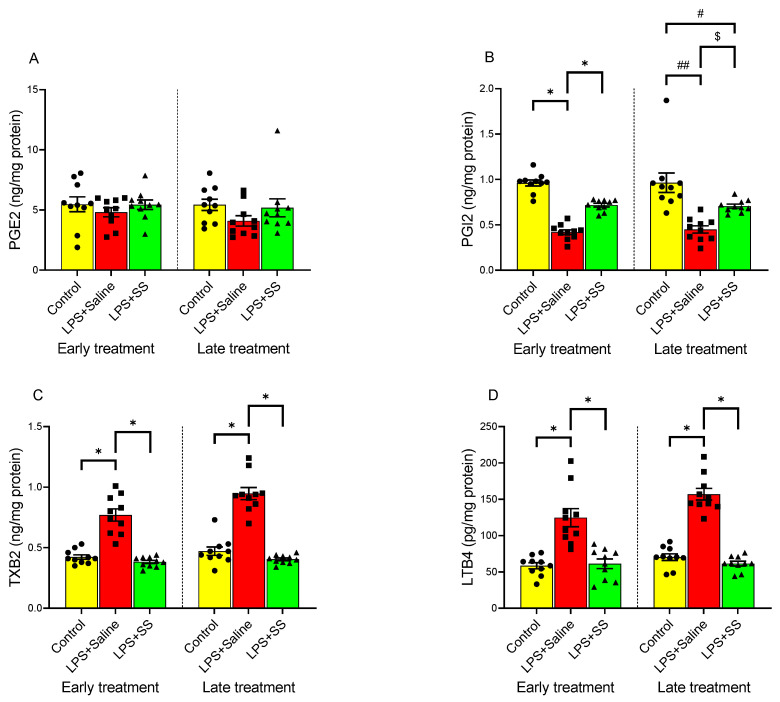
The figure shows the content of PGE2 (ng/mg protein) (**A**), PGI2 (ng/mg protein) (**B**), TXB2 (ng/mg protein) (**C**), and LTB4 (pg/mg protein) (**D**) in the gastric mucosa of male Wistar rats after 30 min of LPS administration + 120 min of SS treatment (early treatment; total procedure duration of 150 min) or 120 min of LPS administration + 120 min of SS treatment (late treatment; total procedure duration of 240 min). Treated animals received LPS + Saline (red columns) or LPS + SS (green columns). Control animals received only saline (yellow columns). (*) *p* < 0.01 LPS + Saline vs. the rest of the groups; (#) *p* < 0.05 LPS + SS or (##) *p* < 0.01 LPS + Saline vs. the control groups; ($) *p* < 0.05 LPS + SS vs. LPS + Saline.

PGE2, PGI2, TXB2, and LTB4 play key roles in the gastric mucosa, regulating inflammation, gastric defense, and repair mechanisms in the mucosa. PGE2 promotes mucosal protection by stimulating mucus and bicarbonate secretion, enhancing blood flow, and inhibiting acid production, while PGI2 helps maintain gastric mucosal integrity by promoting vasodilation and inhibiting platelet aggregation. As shown in Figure 3A, no significant changes were observed in the concentration of PGE2 in the gastric mucosa compared to the control group after LPS administration, both in the early treatment group (control group 5.47 ± 0.62 vs. LPS + Saline 4.81 ± 0.37 ng/mg protein) and the late treatment group (control group 5.43 ± 0.47 vs. LPS + Saline 4.097 ± 0.43 ng/mg protein). However, intraperitoneal injection of LPS induced a significant (*p* < 0.01) decrease in gastric tissue PGI2 production (Figure 3B), both at 150 min and 240 min, as well as a significant (*p* < 0.01) increase in tissue TXB2 (Figure 3C) and LTB4 (Figure 3D) production compared to the control group, in both the early and late treatment groups.

Treatment with SS did not significantly alter the effect of LPS administration in PGE2 gastric mucosa content (LPS + SS 5.44 ± 0.39 vs. LPS + Saline 4.81 ± 0.37 ng/mg protein, early treatment; and LPS + SS 5.18 ± 0.75 vs. LPS + Saline 4.097 ± 0.43 ng/mg protein, late treatment; Figure 3A).

In contrast, SS treatment, both 30 min (LPS + SS 0.72 ± 0.0197 ng/mg protein vs. LPS + Saline 0.42 ± 0.03, *p* < 0.01) and 120 min (LPS + SS 0.706 ± 0.0215 ng/mg protein vs. LPS + Saline 0.45 ± 0.04 ng/mg protein, *p* < 0.05) after LPS administration (total procedure duration of 120 or 240 min, respectively), reversed the LPS-induced reduction in PGI2 production. However, the increase was significantly lower in the late treatment group (early treatment control group 0.96 ± 0.034 ng/mg protein vs. LPS + SS 0.72 ± 0.0197 ng/mg protein, *p* < 0.01; and late treatment control group 0.96 ± 0.11 ng/mg protein vs. LPS + SS 0.706 ± 0.0215 ng/mg protein, *p* < 0.05; Figure 3B).

TXB2 is primarily involved in platelet aggregation and vasoconstriction, but is also linked to inflammation and gastric injury under certain conditions. Similar to the results observed for PGI2, SS administration completely reversed the effect of LPS on TXB2 production in gastric mucosal tissue, both at 150 min (LPS + SS 0.38 ± 0.013 vs. LPS + Saline 0.77 ± 0.05 ng/mg protein, *p* < 0.01) and at 240 min (LPS + SS 0.404 ± 0.011 vs. LPS + Saline 0.947 ± 0.05 ng/mg protein, *p* < 0.01). No significant differences were observed between the LPS + SS group and the control group (early treatment control group 0.42 ± 0.02 ng/mg protein vs. LPS + SS 0.38 ± 0.013 ng/mg protein, *p* < 0.01; and late treatment control group 0.47 ± 0.035 ng/mg protein vs. LPS + SS 0.404 ± 0.011 ng/mg protein, *p* < 0.01; Figure 3C).

The effect of LPS and SS on LTB4 production is shown in Figure 3D. This arachidonic acid metabolite plays a role in inflammation by attracting immune cells (e.g., neutrophils) to sites of injury, contributing to mucosal damage during inflammation. As for TXB2, both early and late treatment with SS significantly counteracted the increase in LTB4 seen as a result of intraperitoneal LPS injection (early treatment LPS + SS 61.10 ± 6.625 vs. LPS + Saline 124.6 ± 12.40 pg/mg protein, *p* < 0.01; late treatment LPS + SS 61.43 ± 3.233 vs. LPS + Saline 156.9 ± 7.969 pg/mg protein, *p* < 0.01). Once again, no significant differences were found when comparing the results obtained in the control group (early treatment control group 58.41 ± 4.18 pg/mg protein; late treatment control group 70.30 ± 4.55 pg/mg protein) with the SS-treated groups (early treatment 61.10 ± 6.625 pg/mg protein; late treatment 61.43 ± 3.233 pg/mg protein).

LPS administration significantly decreased PGI2 levels and increased TXB2 and LTB4 production, indicating heightened inflammation and gastric injury. SS treatment reversed these effects. PGE2 levels in response to LPS-induced inflammation were not significantly altered by SS treatment.

### 3.4. Effect of Early and Late SS Treatment on NO, NOS Activity, and Nitrosothiols in Gastric Mucosa After LPS Administration

NO is a signaling molecule that helps maintain mucosal integrity by promoting vasodilation, enhancing mucus production, and protecting against gastric acid damage. Therefore, endogenous NO may play a role in protecting the gastric mucosa from stimuli that lead to ulceration. NOS produces NO. During inflammation, iNOS is activated and this may result in excess NO production and contribute to gastric injury. Nitrosothiols, formed by the reaction of NO with thiol groups, may protect the mucosal lining, but excessive formation during inflammation may also contribute to injury and disease progression.

**Figure 4:**
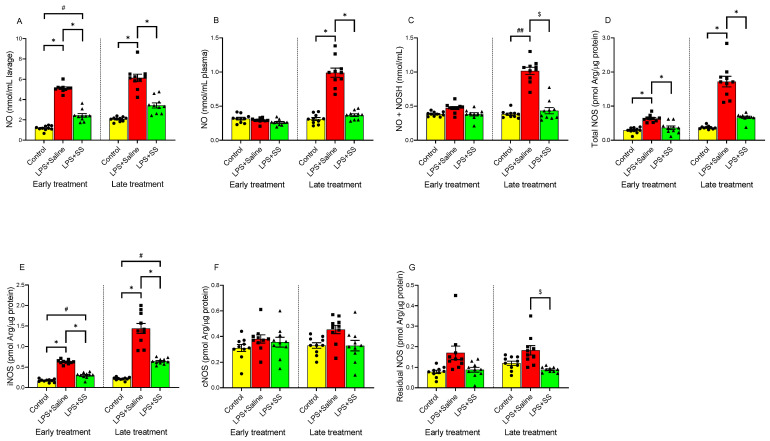
The figure shows NO concentration in gastric lavage (nmol/mL lavage) (**A**), and plasma (nmol/mL plasma) (**B**), nitrosothiol (NO + NOSH) content (nmol/mL) (**C**), total NOS activity (pmol Arg/µg protein) (**D**), iNOS activity (pmol Arg/µg protein) (**E**), cNOS activity (pmol Arg/µg protein) (**F**), and residual NOS (pmol Arg/µg protein) (**G**), after 30 min of LPS administration + 120 min of SS treatment (early treatment; total procedure duration of 150 min) or 120 min of LPS administration + 120 min of SS treatment (late treatment; total procedure duration of 240 min). Treated animals received LPS + Saline (red columns) or LPS + SS (green columns). Control animals received only saline (yellow columns). (*) *p* < 0.01 LPS + Saline vs. the rest of the groups; (#) *p* < 0.05 LPS + SS or (##) *p* < 0.01 LPS + Saline vs. the control groups; ($) *p* < 0.05 LPS + SS vs. LPS + Saline.

In the gastric lavage fluid (Figure 4A), LPS significantly increased NO levels (*p* < 0.01) at both 150 min (5.11 ± 0.125 nmol/mL lavage) and 240 min (6.13 ± 0.36 nmol/mL lavage) compared to their respective controls (1.175 ± 0.074 nmol/mL lavage and 2.06 ± 0.07 nmol/mL lavage). Regarding plasma NO levels (Figure 4B), LPS administration did not significantly alter these values at 150 min (0.2913 ± 0.011 nmol/mL plasma) compared to the control (0.316 ± 0.019 nmol/mL plasma). However, at 240 min, a significant increase (*p* < 0.01) in plasma NO levels was observed following intraperitoneal LPS injection (LPS + Saline 0.99 ± 0.067 vs. control 0.31 ± 0.02 nmol/mL plasma). A similar effect was observed in nitrosothiol (NO + NOSH) production in the gastric mucosa (Figure 4C), as seen in plasma, with no significant differences in the early treatment group (control 0.38 ± 0.01 vs. LPS + Saline 0.472 ± 0.02 nmol/mL plasma) but significant differences in the late treatment group (control 0.38 ± 0.017 vs. LPS + Saline 1.017 ± 0.05 nmol/mL, *p* < 0.01).

The administration of SS in the early treatment group partially blocked the LPS-induced changes in NO levels in gastric lavage (2.43 ± 0.18 vs. 5.11 ± 0.125 nmol/mL lavage, *p* < 0.05; Figure 4A), but no significant changes were observed in plasma (LPS + SS 0.261 ± 0.013 vs. early treatment control group 0.316 ± 0.019 nmol/mL plasma; Figure 4B) or in nitrosothiol (NO + NOSH) production (LPS + SS 0.38 ± 0.02 vs. early treatment control group 0.38 ± 0.01 nmol/mL plasma; Figure 4C).

In the late treatment group, administering SS 120 min after LPS (total procedure duration of 240 min) completely blocked the effects of LPS in gastric lavage (LPS + SS 3.42 ± 0.252 vs. LPS + Saline 6.13 ± 0.36 nmol/mL lavage, *p* < 0.01), plasma (LPS + SS 0.368 ± 0.0196 vs. LPS + Saline 0.99 ± 0.067 nmol/mL plasma, *p* < 0.01), and nitrosothiol (NO + NOSH) production (LPS + SS 0.433 ± 0.046 vs. LPS + Saline 1.017 ± 0.05 nmol/mL, *p* < 0.05), as shown in Figure 4A–C.

LPS administration significantly increased (*p* < 0.01) total NOS activity (Figure 4D) as well as iNOS activity (Figure 4E) in both early and late treatment groups. However, no significant differences were observed in cNOS (Figure 4F) or residual NOS (Figure 4G) activities.

Treatment with SS reversed the LPS-induced increase in total NOS activity (Figure 4D) at both 150 min (LPS + SS 0.365 ± 0.05 vs. LPS + Saline 0.64 ± 0.03 pmol Arg/µg protein, *p* < 0.01) and 240 min (LPS + SS 0.671 ± 0.036 vs. LPS + Saline 1.72 ± 0.16 pmol Arg/µg protein, *p* < 0.01), with no significant differences compared to controls (early treatment control group 0.29 ± 0.026 vs. LPS + SS 0.365 ± 0.05 pmol Arg/µg protein; and late treatment control group 0.37 ± 0.016 vs. LPS + SS 0.671 ± 0.036 pmol Arg/µg protein).

For iNOS activity (Figure 4E), SS treatment, both early and late, partially blocked the LPS-induced increase (LPS + SS 0.299 ± 0.022 vs. LPS + Saline 0.626 ± 0.016 pmol Arg/µg protein, *p* < 0.01, early treatment; and LPS + SS 0.642 ± 0.022 vs. LPS + Saline 1.44 ± 0.123 pmol Arg/µg protein, *p* < 0.01, late treatment), with significant differences still remaining compared to controls (early treatment control group 0.17 ± 0.012 vs. LPS + SS 0.299 ± 0.022 pmol Arg/µg protein, *p* < 0.05; and late treatment control group 0.215 ± 0.011 vs. LPS + SS 0.642 ± 0.022 pmol Arg/µg protein, *p* < 0.05).

No significant differences were observed in cNOS levels (Figure 4F) with early SS treatment (LPS + SS 0.355 ± 0.04 vs. LPS + Saline 0.38 ± 0.032 pmol Arg/µg protein; control 0.31 ± 0.028 vs. LPS + SS 0.355 ± 0.04 pmol Arg/µg protein) or late SS treatment (LPS + SS 0.328 ± 0.04 vs. LPS + Saline 0.454 ± 0.032 pmol Arg/µg protein; control 0.329 ± 0.02 vs. LPS + SS 0.328 ± 0.04 pmol Arg/µg protein).

Regarding residual NOS (Figure 4G), the results were similar to those for cNOS in the early treatment group, with no significant differences between the LPS + SS group (0.088 ± 0.012 pmol Arg/µg protein) and the LPS + Saline group (0.17 ± 0.033 pmol Arg/µg protein), nor between the LPS + SS group and control (0.078 ± 0.008 pmol Arg/µg protein). Conversely, in the late treatment group, a significant reduction in residual NOS levels was observed (LPS + SS 0.0886 ± 0.004 vs. LPS + Saline 0.182 ± 0.0229 pmol Arg/µg protein, *p* < 0.05), with no significant differences between the LPS + SS group and control (0.12 ± 0.01 pmol Arg/µg protein).

In conclusion, LPS administration resulted in a significant rise in NO levels in gastric lavage fluid, plasma, and nitrosothiol production at 240 min. SS treatment attenuated this increase. Additionally, LPS elevated total NOS and iNOS activities, but SS treatment reduced them to near control levels. No significant changes were observed in cNOS or residual NOS activity, except for a reduction in residual NOS in the late treatment group with SS.

### 3.5. Effect of Early and Late SS Treatment on CO in Gastric Mucosa After LPS Administration

**Figure 5:**
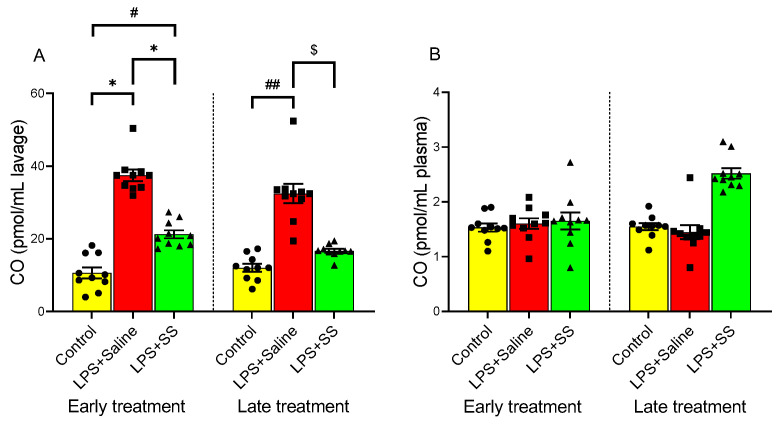
The figure shows CO concentration in gastric lavage (pmol/mL lavage) (**A**), and plasma (pmol/mL plasma) (**B**), after 30 min of LPS administration + 120 min of SS treatment (early treatment; total procedure duration of 150 min) or 120 min of LPS administration + 120 min of SS treatment (late treatment; total procedure duration of 240 min). Treated animals received LPS + Saline (red columns) or LPS + SS (green columns). Control animals received only saline (yellow columns). (*) *p* < 0.01 LPS + Saline vs. the rest of the groups; (#) *p* < 0.05 LPS + SS or (##) *p* < 0.01 LPS + Saline vs. the control groups; ($) *p* < 0.05 LPS + SS vs. LPS + Saline.

CO is a signaling molecule involved in inflammation. Figure 5 illustrates the effect of LPS and SS on CO levels in gastric lavage fluid (Figure 5A) and plasma (Figure 5B). LPS induced a significant increase in CO levels in gastric lavage fluid compared to the control group, both in the early treatment group (LPS + Saline 37.5 ± 1.61 vs. control 10.65 ± 1.51 pmol/mL lavage, *p* < 0.01) and in the late treatment group (LPS + Saline 32.45 ± 2.65 vs. 12.04 ± 1.093 pmol/mL lavage, *p* < 0.01).

Similar to what was observed with NO, no significant differences were detected in plasma CO levels following LPS administration at 150 min (control 1.53 ± 0.076 vs. LPS + Saline 1.604 ± 0.096 pmol/mL plasma) or 240 min (control 1.548 ± 0.065 vs. LPS + Saline 1.444 ± 0.128 pmol/mL plasma). Likewise, no significant changes were observed with SS treatment (LPS + Saline 1.604 ± 0.096 vs. LPS + SS 1.651 ± 0.156 pmol/mL plasma, early treatment group; and LPS + Saline 1.444 ± 0.128 vs. LPS + SS 2.519 ± 0.095 pmol/mL plasma, late treatment group). However, SS treatment administered 30 min after LPS (early treatment group; total procedure duration of 150 min) significantly reduced (*p* < 0.01) CO concentrations in gastric lavage fluid (LPS + SS 21.26 ± 1.115 vs. LPS + Saline 37.5 ± 1.61 pmol/mL lavage), partially blocking the effect of LPS. Significant differences were still observed compared to the control group (LPS + SS 21.26 ± 1.115 vs. 10.65 ± 1.51 pmol/mL lavage, *p* < 0.05). When SS was administered 120 min after LPS (late treatment group; total procedure duration of 240 min), it also significantly reduced (*p* < 0.05) CO concentrations in gastric lavage fluid (LPS + SS 16.72 ± 0.561 vs. LPS + Saline 32.45 ± 2.65 pmol/mL lavage), with no significant differences compared to the control group.

In summary, LPS significantly increased CO levels in gastric lavage fluid and SS treatment reduced its levels. Plasma CO levels remained unchanged.

### 3.6. Effect of Early and Late SS Treatment on cGMP in Gastric Mucosa After LPS Administration

**Figure 6:**
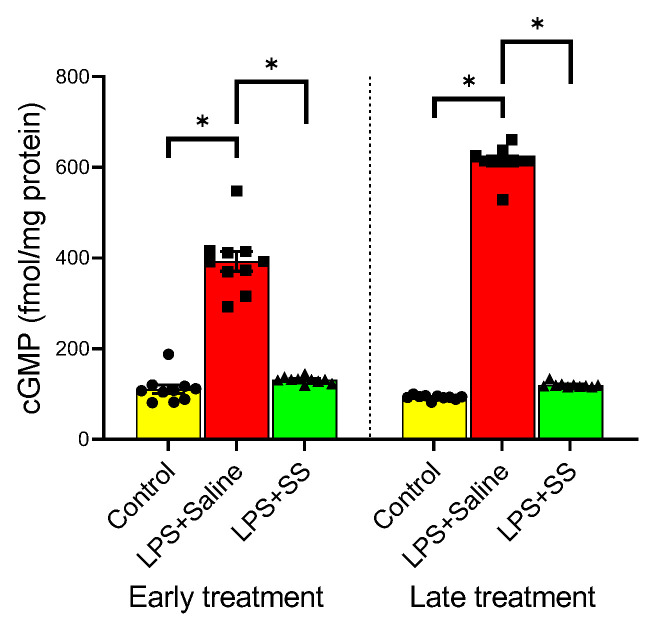
The figure shows cGMP content (fmol/mg protein) after 30 min of LPS administration + 120 min of SS treatment (early treatment; total procedure duration of 150 min) or 120 min of LPS administration + 120 min of SS treatment (late treatment; total procedure duration of 240 min). Treated animals received LPS + Saline (red columns) or LPS + SS (green columns). Control animals received only saline (yellow columns). (*) *p* < 0.01 LPS + Saline vs. the rest of the groups.

cGMP is a second messenger involved in various physiological processes, including vasodilation, regulation of smooth muscle relaxation, and blood flow. Under physiological conditions, NO exerts its actions by activating the soluble fraction of guanylate cyclase, leading to an increase in the concentration of cGMP, which could even be used as a marker of NO activity. The administration of LPS significantly increased cGMP levels (Figure 6) at both 150 and 240 min (LPS + Saline 392.6 ± 21.69 vs. early treatment control group 110.8 ± 9.624 fmol/mg protein, *p* < 0.01; and LPS + Saline 613.8 ± 10.68 vs. late treatment control group 92.8 ± 1.512 fmol/mg protein, *p* < 0.01). This increase was completely reversed with the early treatment (LPS + SS 131.3 ± 2.182 vs. LPS + Saline 392.6 ± 21.69 fmol/mg protein, *p* < 0.01) as well as with the late treatment (LPS + SS 119.8 ± 1.662 vs. LPS + Saline 613.8 ± 10.68 fmol/mg protein, *p* < 0.01) of SS. Thus, both SS treatments effectively counteracted the elevated cGMP levels induced by LPS.

## 4. Discussion

The defense of the gastric mucosa involves a complex interplay of various elements, including mucus, bicarbonate, and surface-active phospholipids. Surface-active phospholipids and their associated fatty acids, synthesized and secreted by gastric surface cells, play a crucial role in the mucosal barrier due to their hydrophobic properties, which significantly contribute to its effectiveness [21]. Disruptions to these protective factors can render the gastric mucosa more vulnerable to injury, particularly under pathological conditions such as sepsis.

SS, a cyclic peptide secreted by D cells in the gastric and intestinal mucosa, as well as by Langerhans islet cells and enteric neurons, has been widely recognized for its regulatory and protective roles in gastrointestinal pathology. Previous studies have demonstrated the efficacy of SS and its analogs in maintaining mucosal integrity, reducing sepsis-induced intestinal barrier dysfunction [22], and mitigating recurrent gastrointestinal bleeding due to gastrointestinal angiodysplasias [23].

PC plays a key role in maintaining the gastric mucosal barrier’s hydrophobicity and shields it from harmful agents [4]. Similarly, PC has been found to support mucosal integrity during inflammatory conditions, including LPS-induced injury in animal models, reinforcing its protective role in the gastrointestinal system [24].

In the present study, SS exerted a protective effect against LPS-induced gastric mucosal injury by restoring phospholipid synthesis, particularly PC and DPPC, which represents approximately 30% of gastric PC. This preservation of phospholipid levels contributed to the maintenance of the hydrophobic properties of the gastric mucosal barrier, reinforcing previous findings on the cytoprotective role of SS in gastroduodenal ulceration [25]. Notably, decreased levels of SS have been associated with an increased risk of gastric ulcer formation, further supporting its critical function in mucosal defense [26].

PLA2 in sepsis-related gastric mucosal injury is another crucial aspect of our study. Elevated serum PLA2 activity has been implicated in the pathogenesis of sepsis caused by Gram-negative bacteria, contributing to neutrophil aggregation, vascular obstruction, and inflammatory cascades, which may result in exacerbating the harmful impact of sepsis. This could partially explain the decrease in the concentration of gastric mucosal PC observed in our LPS-challenged rats. Our results demonstrate that SS inhibited PLA2 activity, potentially accounting for its protective effects. Given that PLA2 activation requires calcium, and SS has been shown to reduce intracellular calcium levels, it is plausible that SS exerted its inhibitory effect on PLA2 through calcium modulation [27]. This finding aligns with previous studies suggesting that SS analogues possess anti-inflammatory properties, further underscoring their potential therapeutic applications [28].

Moreover, it was observed that SS significantly reduced MPO and MDA levels, markers of leukocyte infiltration, and lipid peroxidation, respectively. The LPS-induced increase in MPO activity suggests that infiltrating leukocytes contribute to oxidative stress and mucosal injury. The ability of SS to mitigate this response is consistent with its known immunomodulatory properties, including inhibition of neutrophil, macrophage, and lymphocyte responses [29]. The correlation between MPO and MDA levels further suggests that lipid peroxidation may result from leukocyte-derived free radicals, implicating oxidative stress as a key mediator of LPS-induced gastric damage. SS’s anti-inflammatory effects, including inhibition of NF-κB activation [22] and downregulation of pro-inflammatory cytokines, modulate intestinal inflammatory responses [30], which may contribute to its ability to preserve gastric mucosal integrity under septic conditions. The elevated tissue MPO activity, indicative of leukocyte infiltration in gastric tissue, observed in the rats injected with LPS, raised the question of whether these cells contribute to gastric injury or are merely a consequence of it. Although it is challenging to distinguish between these two possibilities, the strong correlation between MPO and MDA levels suggests that part of the lipid peroxidation in the stomach may be due to infiltrating leukocytes, given their efficient free radical production system [31]. Lipid peroxidation could occur both within the leukocytes themselves and in the adjacent cells.

LPS enhances macrophage production of arachidonic acid metabolites via the cyclooxygenase pathway, generating prostaglandins and thromboxanes, or the lipoxygenase pathway, leading to leukotriene synthesis, potentially mediated by PLA2 activation through cytokines or other mediators. In our study, SS administration counteracted the effects of LPS, leading to an increase in PGI2 levels while reducing TXB2 and LTB4 production, both of which are associated with mucosal damage. These findings reinforce existing literature that suggests prostaglandins mediate SS’s gastroprotective effects [32,33], potentially through the activity of sulfhydryl groups or by stimulating endogenous prostaglandin secretion, although this still requires further investigation [34]. These defensive mechanisms are thought to involve inhibition of acid secretion and modulation of gastric vasculature. The efficacy of SS in protecting the gastric mucosa from injury in various experimental models, including endotoxemia models, is, however, not solely attributed to the inhibition of acid secretion. SS may exert its protective effects through mechanisms that are not yet fully understood but are believed to involve neuropeptides, such as substance P and VIP, as well as the inhibition of mucosal leukotriene production, as demonstrated in an experimental model of ethanol-induced mucosal injury in rats [35]. However, our data showed no significant changes in PGE2 concentrations, which is consistent with previous research indicating that SS’s inhibition of gastric acid secretion may occur independently of PGE2 production, likely through the suppression of gastrin secretion [36].

Another key aspect of the protective mechanism of SS involves its regulation of NO signaling. Thus, it has been documented that SS exerts gastroprotective effects and aids in restoring gastric mucosal blood flow during exposure to the harmful effects of ethanol through mechanisms dependent on NO generation [37]. In endotoxemia, vascular smooth muscle tone is diminished, resulting in hypotension despite high cardiac output. NO seems to play a key role in the vasodilation observed in sepsis models [38]. Vasodilation is an active process involving the production of cyclic adenosine monophosphate (cAMP) and cGMP in the cytosol, which leads to the relaxation of vascular smooth muscle tone. Guanylate cyclase, the enzyme responsible for cGMP synthesis, is activated by NO [39]. It has been suggested that the hypotension seen during sepsis may be attributed to excessive NO production, which, through cGMP, exerts its effects on endothelial smooth muscle cells [40]. Like NO, CO also shares similar properties, such as the ability to activate soluble guanylate cyclase, leading to an increase in cGMP levels [41]. In the present study, SS inhibited NOS activity (total and inducible), reducing the observed LPS-induced overproduction of NO, CO, and cGMP, which may be implicated in sepsis-related gastric mucosal injury. This supports the notion that SS exerts its protective effects by modulating the NO/cGMP pathway [42], a hypothesis further supported by studies showing colocalization of SS receptors with NOS or NADPH-diaphorase (a marker for NOS) [43,44,45]. The ability of SS to restore vascular homeostasis and reduce excessive vasodilation suggests a broader therapeutic potential in sepsis management, particularly in mitigating systemic inflammatory responses. Further research should identify the specific factors involved in each stage of the cascade related to the role of NO/cGMP and clarify the SS−NO interaction in the physiological and protective effects of SS on the gastric mucosa.

In summary, our findings provide substantial evidence that SS plays a critical role in mitigating LPS-induced gastric mucosal injury through multiple mechanisms, including the restoration of phospholipid synthesis, modulation of arachidonic acid metabolism, inhibition of NO and PLA2 activity, and suppression of oxidative stress and leukocyte infiltration. These results emphasize the importance of SS in gastrointestinal protection and suggest its potential as a therapeutic agent in sepsis-related gastric injury. Future studies should aim to elucidate the precise molecular interactions underlying SS’s protective effects, particularly its interplay with the NO/cGMP and PLA2 pathways, to further explore its clinical applications in managing sepsis-induced gastrointestinal dysfunction.

## 5. Conclusions

LPS significantly inhibited the synthesis of PC and its disaturated form, DPPC, in the gastric mucosa. These changes were accompanied by increased MPO activity and elevated levels of MDA, TXB2, and LTB4. In contrast, LPS reduced PGI2 mucosal levels, with no significant changes in PGE2. Additionally, LPS elevated NO and CO levels in the gastric lavage fluid. This was associated with enhanced iNOS activity and increased cGMP levels in the mucosa. Both NO and CO are diffusible gases, and their elevated levels in gastric lavage may reflect increased plasma concentrations enabling their movement into the gastric lumen. However, we believe this rise was driven by local production, as plasma levels remained largely unchanged.

Treatment with SS effectively countered the effects induced by bacterial LPS. Our results suggest that Gram-negative bacterial sepsis compromises the gastric mucosal barrier by reducing its hydrophobic phospholipid content. Furthermore, they indicate that SS may play a protective role in gastric lesions secondary to sepsis by restoring PC production and suppressing the generation of other potential harmful mediators. The disruption of the gastric mucosal barrier in sepsis may, at least partly, result from altered NO and CO production, leading to guanylate cyclase activation and elevated cGMP levels.

Although the primary focus of the study was to assess the effects of SS on the gastric mucosal barrier in the context of LPS-induced sepsis, quantification of SS in blood plasma as well as using fluorescence-activated cell sorting (FACS) to evaluate immune cell alterations following LPS treatment with or without SS would have provided significant insights into the modulation of immune cell populations and the inflammatory response under these conditions. Future studies incorporating FACS analysis could elucidate the immunomodulatory effects of SS in this model.

## Data Availability

The data that support the findings of this study are available from the corresponding author upon reasonable request.
